# Glycolipid Metabolic Disorders, Metainflammation, Oxidative Stress, and Cardiovascular Diseases: Unraveling Pathways

**DOI:** 10.3390/biology13070519

**Published:** 2024-07-12

**Authors:** Enzo Pereira de Lima, Renato Cesar Moretti, Karina Torres Pomini, Lucas Fornari Laurindo, Kátia Portero Sloan, Lance Alan Sloan, Marcela Vialogo Marques de Castro, Edgar Baldi, Bruna Fidencio Rahal Ferraz, Eliana de Souza Bastos Mazuqueli Pereira, Virgínia Maria Cavallari Strozze Catharin, Carolina Haber Mellen, Flávia Cristina Castilho Caracio, Caio Sérgio Galina Spilla, Jesselina F. S. Haber, Sandra Maria Barbalho

**Affiliations:** 1 Department of Biochemistry and Pharmacology, School of Medicine, Universidade de Marília (UNIMAR), Marília 17525-902, SP, Brazil; enzopereiralima@outlook.com (E.P.d.L.);; 2 Postgraduate Program in Structural and Functional Interactions in Rehabilitation, School of Medicine, Universidade de Marília (UNIMAR), Marília 17525-902, SP, Brazil; 3 Department of Biochemistry and Pharmacology, School of Medicine, Faculdade de Medicina de Marília (FAMEMA), Marília 17525-902, SP, Brazil; 4 Texas Institute for Kidney and Endocrine Disorders, Lufkin, TX 75904, USA; 5 Department of Internal Medicine, University of Texas Medical Branch, Galveston, TX 77555, USA; 6 Department of Odontology, Universidade de Marília (UNIMAR), Marília 17525-902, SP, Brazil; 7 Department of Internal Medicine, Irmandade da Santa Casa de Misericórdia de São Paulo (ISCMSP), São Paulo 01221-010, SP, Brazil; 8 Department of Psychology, School of Psychology, Universidade de Marília (UNIMAR), Marília 17525-902, SP, Brazil; 9 Charity Hospital, UNIMAR (HBU), Universidade de Marília, UNIMAR, São Paulo 17525-160, SP, Brazil

**Keywords:** glycolipid metabolic disorders, cardiovascular diseases, atherogenesis, hyperglycemia, dyslipidemia, metainflammation, oxidative stress

## Abstract

**Simple Summary:**

Glycolipid metabolic disorders (GLMDs) result from imbalances in glycolipid levels, leading to various health issues, including obesity, diabetes, liver problems, nerve and muscle complications, and cardiovascular and kidney diseases. This study explores the connection between GLMDs, oxidative stress, and chronic inflammation, which exacerbate these conditions. GLMD originates from disruptions in glucose and fat metabolism, often associated with hormone regulation and insulin resistance. These disruptions cause the accumulation of harmful molecules, triggering inflammation in multiple organs. Key molecules, such as advanced glycation end products (AGEs) and sphingosine-1-phosphate (S1P), play significant roles in this process. Understanding these relationships is essential for developing better treatments, reducing illness and mortality rates, lowering healthcare costs, and improving quality of life.

**Abstract:**

Glycolipid metabolic disorders (GLMDs) are various metabolic disorders resulting from dysregulation in glycolipid levels, consequently leading to an increased risk of obesity, diabetes, liver dysfunction, neuromuscular complications, and cardiorenal vascular diseases (CRVDs). In patients with GLMDs, excess caloric intake and a lack of physical activity may contribute to oxidative stress (OxS) and systemic inflammation. This study aimed to review the connection between GLMD, OxS, metainflammation, and the onset of CRVD. GLMD is due to various metabolic disorders causing dysfunction in the synthesis, breakdown, and absorption of glucose and lipids in the body, resulting in excessive ectopic accumulation of these molecules. This is mainly due to neuroendocrine dysregulation, insulin resistance, OxS, and metainflammation. In GLMD, many inflammatory markers and defense cells play a vital role in related tissues and organs, such as blood vessels, pancreatic islets, the liver, muscle, the kidneys, and adipocytes, promoting inflammatory lesions that affect various interconnected organs through their signaling pathways. Advanced glycation end products, ATP-binding cassette transporter 1, Glucagon-like peptide-1, Toll-like receptor-4, and sphingosine-1-phosphate (S1P) play a crucial role in GLMD since they are related to glucolipid metabolism. The consequences of this is system organ damage and increased morbidity and mortality.

## 1. Introduction

Glycolipid metabolic disorder (GLMD) is a condition related to the environment, genetics, dietary patterns, psychiatric disorders, and other factors, culminating in lipid and glucose metabolism disorders. GLMD is a massive global health problem due to its association with people’s lifestyles, and leads to harmful effects on human health, such as CVRD and its many complications [1]. The growing and worrying increase in obesity anticipates a substantial challenge in preventing chronic diseases and reducing global deaths in future public health systems [2,3]. However, it is challenging to promote the improvement in the health conditions of all patients, especially in contemporary societies where overnutrition is prevalent and the lack of exercise is a reality in both developed and developing countries [4,5].

**Figure 1:**
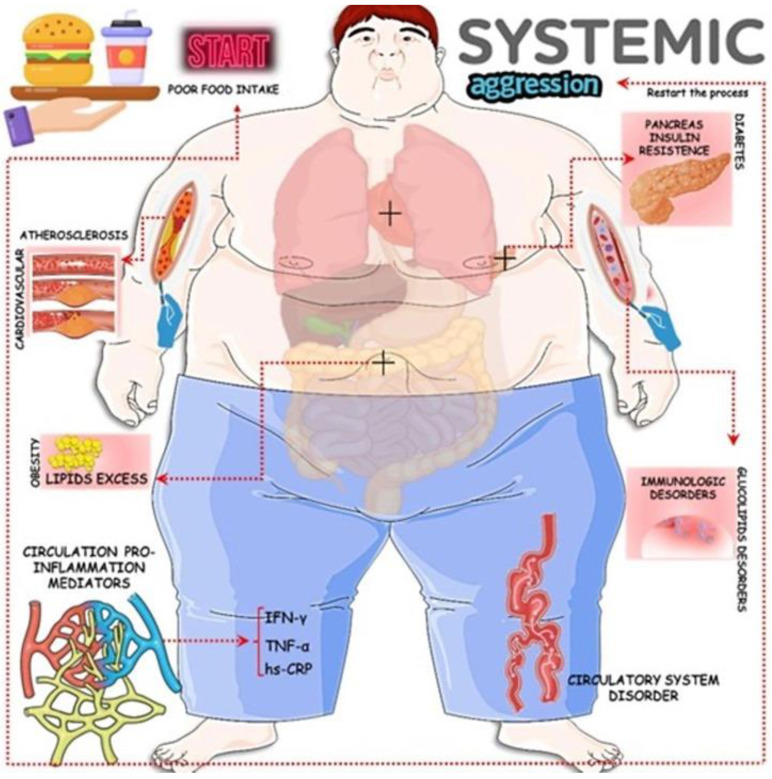
Glycolipid metabolic disorders are related to unhealthy habits, leading to alterations in the body’s metabolism, initiating inflammatory processes and oxidative stress, and leading to several metabolic conditions such as insulin resistance, diabetes, obesity, metabolic syndrome, and CVD. In summary, this image makes explicit the systemic aggression and its complications. CVD: cardiovascular disease; hs-CRP: hs-C reactive protein; IFN-γ: Interferon-γ; TNF-α: tumor necrosis factor-α.

**Figure 2:**
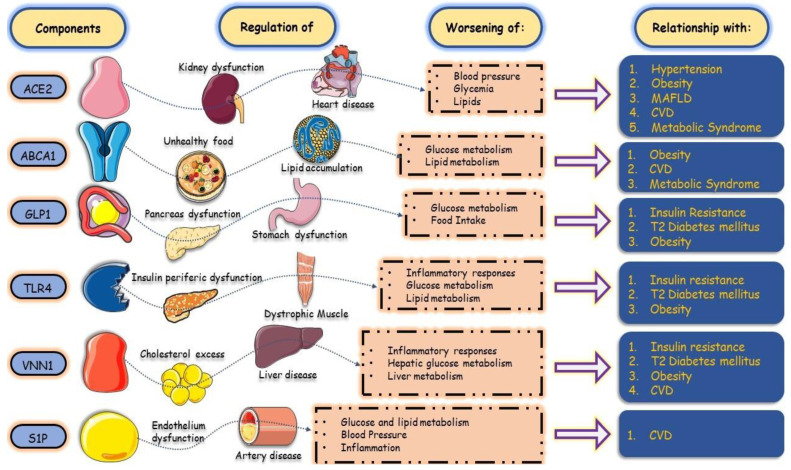
The relationship between chemical mediators in glycolipid metabolic disorders and the affected systems. The imbalance in glycolipids promotes a systemic inflammatory storm, which can lead to CVD, hormone diseases, and gastrointestinal disorders. ACE-2: Angiotensin-converting enzyme 2; ABCA1: ATP-binding cassette transporter; CVD: cardiovascular disease; GLP-1: Glucagon-like peptide-1; MAFLD: metabolic-associated fatty liver disease; SIP: sphingosine-1-phosphate; TLR4: Toll-like 1296 receptor-4; VNN1: vascular non-inflammatory molecule-1.

The origin of GLMD is related to a variety of metabolic disorders resulting from anomalous processes in the synthesis, breakdown, and absorption of glucose and lipids in the body, resulting in excessive ectopic accumulation of these molecules [6,7]. The mechanisms are mainly through neuroendocrine dysregulation, insulin resistance (IR), oxidative stress (OxS), metainflammation, and dysbiosis of the intestinal flora. Thus, clinical hyperglycemia, dyslipidemia, fatty liver disease, and especially, cardiovascular diseases (CRVDs) can emerge. In GLMD, many inflammatory factors and defense cells play a vital role in related tissues and organs, such as blood vessels, pancreatic islets, the liver, and adipocytes, promoting inflammatory lesions that affect various interconnected organs through their signaling pathways. In this sense, IR, hyperglycemia, and dyslipidemia favor morbidity and mortality in the cardiac system (Figure 1 and Figure 2). In this respect, impairments in glucose regulation, a reflection of insulin deficiency and its resistance in the periphery, and the regulation of lipids result in a chronic disease characterized by an ample blood supply of glucose, that is, Type 2 Diabetes Mellitus (T2DM) [8]. IR is an impaired response to insulin stimulation in target tissues and, when linked to dyslipidemia and chronic low-grade inflammation, particularly in the liver, muscles, and adipose tissue (AT), can lead to metabolic-associated fatty liver disease (MAFLD) and atherosclerosis [9].

Chronic hyperglycemia wreaks damage on the body’s organic systems and fosters cardiac comorbidities because the metabolism of glycolipids is impaired in diabetes and can activate macrophage inflammation, contributing to vascular injury. Hyperglycemia is related to the increase in advanced glycation end product (AGE) levels, which can cause potential damage to several tissues [10,11]. The endogenous formation of AGEs occurs through various mechanisms, including the oxidation of amino acids, lipids, nucleic acids, and sugars and the polyol pathway, which is hyperactive in hyperglycemic states [12,13]. AGEs have significant representatives such as glyoxal, methylglyoxal, 3deoxyglucosone, glycolaldehyde, and glyceraldehyde, which induce pathological effects by modifying the structure and function of organic proteins (cross-linking) or inducing the formation of ROS and inflammatory cytokines, highlighting the connection between chronic inflammatory states, oxidative disorders, and CVD [14,15,16].

Endothelial dysfunction, in addition to the action of AGEs, is induced by the positive regulation of biomolecules such as vascular endothelial growth factor and plasminogen activator inhibitor-1 (PAI-1), leading to neoangiogenesis and increasing the pro-thrombotic state, essential in the formation of CVD [17,18]. AGE-RAGE (AGE receptor) binding triggers various intracellular reactions, resulting in the synthesis of pro-inflammatory cytokines such as interleukin (IL)-6, tumor necrosis factor-α (TNF-α), Transforming Growing Factor (TGF)-β, vascular adhesion molecules like (VCAM-1), intercellular adhesion molecule-1 (ICAM-1), and endothelin-1 (ET-1), which also have a close relationship with CVD [19,20,21,22].

The heart and blood vessels are surrounded by adipose tissue. Due to the impaired metabolism of this tissue, there is a change in the secretory pattern (macrophage M2 converts to M1), leading to the secretion of mediators such as increased resistin and leptin levels, reduced adiponectin secretion, and increases in TNF-α and pro-inflammatory interleukins. These conditions directly or indirectly interfere with the cardiovascular system [23]. In addition, some molecules can be related to GLMD, as mentioned below.

Angiotensin-converting enzyme 2 (ACE2) is a mono-carboxypeptidase that catalyzes a single residue of Angiotensin I (Ang I) and Angiotensin II to form Angiotensin 1–9 (A1–9) and A1–7. Additionally, it plays a critical regulatory role in the absorption of amino acids from the diet in the gastrointestinal tract. ACE2, by generating A1–7, promotes the regulation of the renin–angiotensin–aldosterone system (RAAS). Hypertension, DM2, obesity, and metabolic-associated fatty liver disease (MAFLD) are associated with RAAS disorders and changes in ACE2 expression levels. Therefore, ACE2 dysfunction can promote the progression of metabolic diseases, which include deleterious effects such as vasoconstriction, inflammation, cardiovascular damage, and increased OxS [24,25,26].

ATP-binding cassette transporter 1 (ABCA1) controls lipid and glucose metabolism. It is an essential downstream target gene of peroxisome proliferator-activated receptors (PPARs), whose activation increases ABCA1 expression. This transporter is critical for cholesterol homeostasis in pancreatic β cells and insulin secretion. ABCA1 deficiency in these cells results in impaired function and reduced insulin secretion. Additionally, ABCA1 is the primary mediator of cholesterol efflux in adipocytes. The specific absence of ABCA1 in these adipocytes results in insulin resistance and an obese phenotype. ABCA1 is crucial for proper lipid and glucose metabolism and plays a critical role in the function of pancreatic β cells and adipocytes [27,28,29].

Glucagon-like peptide-1 (GLP-1) is a gastrointestinal hormone produced in enteroendocrine L cells by the differential processing of proglucagon [30]. Researchers have demonstrated that the intestinal microbiota can regulate GLP-1 production through the short-chain fatty acid/G-protein-coupled receptor signaling pathway. This hormone can increase satiety, thereby promoting reduced obesity and defense against cardiovascular events and glycolipid disorders, as it has a direct link to insulin secretion [28,31,32,33].

Toll-like receptor-4 (TLR-4) is a molecule of the innate immune system related to a crucial role in the inflammatory process and regulates glucose and lipid metabolism. Reducing the expression of inflammatory factors linked to TLR4 by interrupting the signaling pathway of this receptor may have a preventive effect on metabolic diseases. TLR-4 activation by lipopolysaccharide (LPS) can stimulate cytosolic phospholipase A2 (cPLA2) activity and AT formation, affecting blood low-density lipoprotein (LDL-c) and high-density lipoprotein (HDL-c) levels. Additionally, inhibiting TLR-4/NF-κB activation can interrupt glucose production in hepatocytes, potentially preventing hyperglycemia [34,35].

Vascular non-inflammatory molecule-1 (VNN1) is a pantetheine hydrolase mercaptoethylamine that regulates OxS, inflammatory responses, cell migration, and glucose metabolism. This molecule is expressed in centrilobular hepatocytes in the liver, and its activity is induced by fasting or insulin resistance, physiologically affecting multiple metabolic pathways. VNN1 plays a relevant role in hepatic lipid metabolism and its overexpression leads to GLMDs that induce the development of chronic diseases [36,37,38].

Certain types of sphingolipids have pathological activity when found in excess in the human body, particularly promoting CVD. Among these, ceramides, sphingolipids, and aberrantly expressed sphingosine-1-phosphate (S1P) are potential risk factors for atrial fibrillation, acute coronary syndrome, arterial hypertension, myocardial ischemia, and especially, atherosclerosis, which is an inflammatory and potentially lethal condition characterized by the formation of atheromatous plaques [39]. These plaques are composed of cholesterol and other lipids in medium and large arteries involving high levels of pro-inflammatory biomarkers, such as TNF-α and interleukins, such as IL-1β, which together with oxidized LDL-c (ox-LDL) drive the formation of ceramides through the hydrolysis of sphingomyelin. Atheromatous plaques induce the formation of IL-6 by macrophages during their formation in the inflammatory process, promoting endothelial injury [40]. Moreover, atheromatous plaques stimulate the liver to produce C-reactive protein (CRP) which is known as the primary systemic inflammatory marker [41]. At the cellular level, GLMD is associated with the pathophysiology of atherosclerosis, such as endothelial dysfunction, i.e., endothelial apoptosis under hyperglycemic conditions [42].

Due to the increasing incidence of GLMD and the intricate mechanisms by which this condition is related to the genesis of different diseases, this study aimed to critically review the physiological pathways and consequences of these metabolic conditions, especially the ones related to CVD.

## 2. Materials and Methods

### 2.1. Databases

MEDLINE-PubMed, EMBASE, and Cochrane databases were searched to build this review. The combination of mesh terms used for this search was glycolipid metabolic disorders, inflammation, metainflammation, oxidative stress, metabolic syndrome, and cardiovascular diseases.

### 2.2. Eligible Criteria and Study Selection

Only studies written and published in English were selected. The inclusion criteria were clinical trials, investigative studies, retrospective studies, reviews, in vitro studies, and animal model studies. The exclusion criteria were articles not in English, case reports, poster presentations, and letters to the editor.

## 3. Ceramides and Other Sphingolipids

The sphingolipids are molecules formed by a hydrophobic and a hydrophilic chain in which the type of fatty acid component determines its species. Among them, it is possible to mention ceramide, monohexosyl ceramide, sphingomyelin, and sphingosine [43]. Some well-known species are ceramides and sphingosine-1-phosphate (S1P). The enzymes involved in converting ceramide into sphingosine and sphingosine into S1P are, respectively, ceramidase and sphingosine kinases [44,45,46]. There are different types of S1P receptors, and S1P exerts potent biological effects such as regulatory signaling in growth, differentiation, inflammation, oxidation, stress response, apoptosis, proliferation, and metabolic and structural function. However, when in excess, the receptors can contribute to the pathogenesis of various conditions such as insulin resistance, which progresses to DM2 (as ceramide accumulation impairs insulin action and promotes the dysfunction of β cells) and causes vascular complications (such as coronary artery disease) and neurodegenerative disorders. As a result, they are implicated in GLMD [47,48,49,50,51].

Sphingosine-1-phosphate (S1P) impairs the survival of pancreatic β cells, leads to insulin resistance both in the liver and skeletal muscle, and worsens inflammation in the adipose tissue. S1P levels are increased in individuals with a high-fat diet in organs such as the liver, adipose tissue, skeletal muscle, the pancreas, and plasma. These mechanisms are understood to accelerate the development of insulin resistance, thus contributing to glycolipid dysregulation [4,52]. Moreover, growing evidence indicates that changes in bioactive sphingolipids, especially S1P and ceramides, are related to heart diseases [29,53]. Some plasma ceramides can indicate major cardiovascular events [54]. Some authors have shown that hemodynamic stress is capable of inducing an early metabolic rewiring regarding de novo endothelial sphingolipid biosynthesis, favoring S1P signaling over ceramides in a protective pathway. NOGO-B (a membrane protein critical in modulating endothelial dysfunction and the pathogenesis of coronary heart diseases) deletion supports the rewiring of sphingolipid metabolism. These findings can provide a possible foundation for sphingolipid-based therapy to limit atherosclerosis [43,55,56,57,58].

Furthermore, ceramide accumulation is associated with muscle disuse [59,60,61]. This sphingolipid accumulates subcellularly within the intramuscular milieu, maximizing its relationship with IR. As a result, it can be inferred that older adults are more likely to accumulate skeletal muscle ceramides than younger adults. A vicious cycle is formed when coupled with a poor diet, culminating in cardiovascular events [62]. The increased plasma delivery of FFAs and triglycerides (TGs) and their oxidation/storage by the heart lead to OxS and significantly contribute to the risk of cardiac dysfunction. Although providing cardiac energy, FFAs serve as substrates for ceramide biosynthesis, which can promote left ventricular dysfunction, heart failure, coronary artery disease (CAD), valvular abnormalities, and hypertension [57,63,64]. Dysregulated lipid pathways, mainly involving ceramides within cardiomyocytes, are considered a potential pathogenic process in developing heart failure with preserved ejection fraction. This metabolic stress can also induce cell cycle arrest, apoptosis, senescence, inflammation, and endothelial dysfunction, culminating in other CVDs [65,66,67,68].

Lipidomics is an effective methodology for investigating lipid metabolism in cells and discovering lipid biomarkers associated with various diseases. Lipidomic analysis of plasma has shown predictive capability in diagnosing several conditions such as cancer, type 2 diabetes, cardiovascular diseases, and systemic lupus erythematosus. This field of study aims to identify specific lipids that can serve as disease markers or molecules with anomalous lipid structures [69]. Advances in imaging techniques, such as coronary artery calcium scoring and coronary CT angiography (CTA), have enhanced our ability to predict risks on a personalized level. In addition to assessing genetic markers and structural changes in arterial walls through imaging, blood biomarkers function as a “liquid biopsy”, revealing causal or sequential markers of atherosclerotic cardiovascular disease risk. Therefore, the use of ‘-omics’ approaches, particularly proteomics and lipidomics, plays a crucial role in this field, enabling early detection of these changes [70]. The two main molecular classes, proteins and lipids, have shown significant potential to enhance the prediction of atherosclerotic cardiovascular disease (ACVD) risk. This approach can uncover new lipids and proteins that may be associated with the risk of this disease. Following the successful identification of candidate biomarkers related to cardiovascular diseases, a targeted approach can be used to quantify the levels of these biomarkers in a high-throughput routine. This has the potential to significantly improve risk stratification for cardiac diseases. The discovery of proteomics allows for the detection of proteins in a specific sample without the need for the prior selection of proteins, thereby expanding the possibilities of identifying relevant markers [71,72].

## 4. The Implications of High Fructose/Glucose Consumption

Fructose is one monosaccharide in honey, fruits, vegetables, and high-fructose corn syrup used to manufacture beverages (soft drinks) and food [73]. High consumption of both glucose and fructose is relevant to the development of GLMD since they are associated with the development of metabolic syndrome risk factors such as hyperglycemia, hypertriglyceridemia, reduced HDL-c, and hypertension. In some cases, the excessive and chronic consumption of fructose leads to increased stress for the endoplasmic reticulum (ER) due to the stimulation of lipid metabolism. This scenario generates inflammation, OxS, and apoptosis in cardiomyocytes. In addition, excess fructose can switch the profile of chemokines with increased production of Fetuin-A, Fibroblast growth factor 21 (FGF-21), Leucocyte cell-derived chemotaxin 2 (LECT2), and Angiopoietin-like protein (ANGPTL), which compromises energy homeostasis, leads to mitochondrial dysfunction, aggravates IR, and contributes to others organ damage [74,75].

It is not new that arterial hypertension is one of the most important predictors of the development of CVD. In this sense, fructose plays a relevant role since sugar consumption acts as an unregulated substrate for lipogenesis, favoring the development of DM and obesity and exacerbating the hypertensive condition. The disorders caused by this uncontrolled consumption of fructose trigger greater accumulation of lipids in the subendothelial layer, promoting the formation of atherosclerotic processes, which are also highly relevant in cardiovascular events [76,77,78].

General excessive sugar consumption, particularly fructose, has also been implicated in the development of metabolic-associated fatty liver disease (MAFLD). Fructose triggers uric acid production and raises blood pressure by enhancing renal sodium and chloride transport, as well as stimulating the sympathetic nervous system and disrupting the balance between vasoconstrictors and vasodilators. Furthermore, high fructose intake amplifies OxS and activates the renin–angiotensin–aldosterone system, thereby contributing to hypertension and, subsequently, CVD [79,80,81,82].

Another mechanism linking fructose to CVD is through copper deficiency (CuD), potentiated by the presence of fructose in the body. Fructose exacerbates CuD-induced cardiac remodeling and the number of intramyocardial lipids. The mechanism by which CuD and fructose promote this cardiac alteration occurs through the inhibition of autophagic flux caused by calcium ion disturbances, resulting in reduced expression of the sarcoplasmic/endoplasmic reticulum Ca^2+^ ATPase 2a (SERCA2a) in cardiomyocytes [83].

Similarly, parental fructose consumption can affect future offspring. A particular study showed that excessive fructose consumption negatively affected parents through metabolic and cardiovascular disorders, which were then echoed in their descendants. The consequences in the next generation were reflected in a lower birth weight, increased blood triglyceride levels, insulin resistance, elevated blood pressure, and impaired baroreflex sensitivity, characterized by reduced bradycardic outcomes and reflex tachycardia [84].

**Figure 3:**
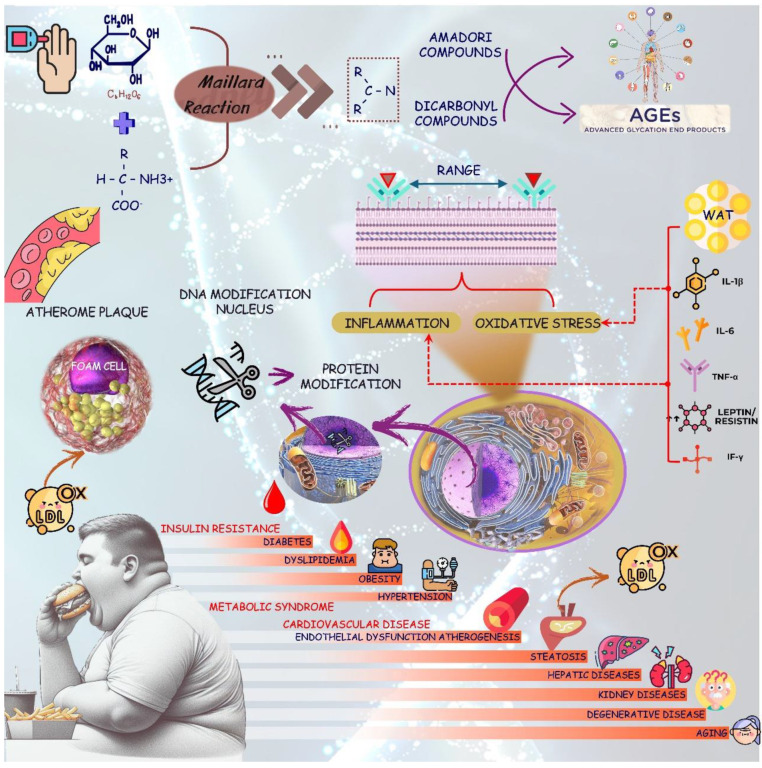
Hyperglycemia is related to the production of AGES. These reactive compounds are related to several metabolic conditions. The increase in unhealthy food intake triggers a rich inflammatory environment, which can result in changes in DNA, protein modifications, insulin resistance, oxidative stress, and inflammation that can be related with several conditions such as obesity, diabetes, metabolic syndrome, and liver, kidney, degenerative, and cardiovascular diseases. AGES: advanced glycation end products; IL-6: Interleukine-6; IL-1β: Interleukine-1β; IF-γ: Interferon-γ; ox-LDL: oxidized low-density lipoprotein; TNF-α: tumor necrosis factor-α; WAT: white adipose tissue.

Under a hyperglycemic environment, glucose reacts with amines from amino acids or proteins, resulting in the formation of reversible Schiff base products, which can lead to the formation of Amadori rearrangement products. After days of successive reactions, the Amadori products undergo dehydration, condensation, fragmentation, rearrangement, and oxidation to finally generate AGEs [85,86]. AGEs produced from glucose can include methylglyoxal-derived hydroimidazolone 1 and argpyrimidine. Fructose can result in AGEs by fructolysis through a phosphorylation process by hexokinase or fructokinase (liver) (fructose → fructose-1P). Further, fructose-1P, through aldolase B, can result in AGEs such as Glyceraldehyde-derived pyridinium compounds and pyrrolopyridinium Lys dimers derived from Glyceraldehyde, argpyrimidine, and others [86,87,88,89]. Figure 3 shows some pathways in the formation of AGEs and the consequences for the human body.

Glycated hemoglobin (HbA1c) is also associated with CVD (ischemic stroke, ischemic heart disease, carotid and coronary atherosclerosis, and hypertension). Additionally, elevated HbA1c levels increase CRP levels, OxS, and blood viscosity, contributing to CVD [90,91,92]. Glycemic alterations, represented by both hyperglycemia and hypoglycemia, can also have an effect in CVD. Hyperglycemia induces the overproduction of ROS and reduces endogenous antioxidant defense through increased release of AGEs, the stimulation of protein kinase C (PKC), and hyperactivity of the hexosamine and sorbitol pathways. In this context, AGEs/ROS are harmful, causing oxidative damage to cellular structures, modulating intracellular signaling pathways, increasing the expression of pro-inflammatory and pro-coagulant factors, inducing apoptosis, and impairing the release of NO. On the other hand, hypoglycemia is also a significant factor in cardiovascular injury through OxS, the release of catecholamines, inflammatory response, platelet activation, pro-thrombotic events, and endothelial dysfunction [93,94,95,96].

## 5. Inflammation and Oxidative Stress: A Path of No Return?

The world is witnessing the growing spread of cardiometabolic diseases due to the population’s lifestyle, which may include diet, sedentary behavior, stress, aging, increasing obesity rates, and other related conditions [97,98,99]. Cardiometabolic disease is a broad term for cardiovascular conditions arising from systemic metabolic changes closely linked to inflammation and OxS. An example is diabetic cardiomyopathy (DCM) induced by elevated glycemic levels and the resulting AGEs [100,101,102]. DCM is a serious complication of both type 1 and type 2 DM characterized by the worsening of myocardial fibrosis, disturbances in both systolic and diastolic functions, and increased mortality. This occurs through mechanisms such as glycolipid toxicity, inflammatory response, and OxS [103]. In a state of IR, fatty acids become the sole energy source for cardiac tissue. Impairments in insulin signaling result in a deficiency in the translocation of the glucose transporter 4 (GLUT4), the primary mediator of insulin-stimulated glucose uptake [104]. Consequently, there is an increase in the uptake and accumulation of lipids in the heart, thereby inducing lipotoxicity and leading to chronic inflammation of cardiac tissue and OxS, causing myocardial damage. However, some argue that the deposition of triglycerides per se is not toxic, but rather, it is the excessive synthesis of reactive oxygen species (ROS) that impairs cardiac function [105,106,107,108].

Free radicals are chemical entities containing at least one unpaired electron in the outer electronic layer, typically rendering them highly reactive. The primary representatives are ROS, which can snatch electrons from any other molecules such as DNA, proteins, and also cell membranes, triggering chain reactions that lead to inflammatory processes, apoptosis, and necrosis [109,110,111,112].

ROS are the primary effectors of injury from OxS and, consequently, can play a crucial role in linking inflammation with CVD [113,114,115]. In this context, mitochondria are the main generators of endogenous ROS through the electron transport chain (ETC) and oxidative phosphorylation, producing byproducts in CVD [116,117,118,119]. In a hypertensive scenario, for example, the inflammatory condition, the product of high production of the superoxide anion (O^2−^) and hydrogen peroxide (H_2_O_2_) by endothelial cells, monocytes, and macrophages promotes OxS that results in vascular dysfunction. Additionally, the RAAS is a crucial regulator of OxS, resulting in inflammatory damage to the vessels [120]. Angiotensin II (Ang II) is produced by the RAAS, promotes vascular inflammation by activating nicotinamide adenine dinucleotide phosphate oxidases (NOXs), and enhances the action of ET-1, leading to an increase in pro-inflammatory mediators and promoting endothelial dysfunction. Excessive ROS produced by NOX 2, the primary isoform in the cardiac system, are primarily responsible for the establishment of hypertension [121,122].

Human epicardial adipose tissue (EAT) is a unique and multifunctional fat compartment of the heart composed of adipocytes, nervous tissue, inflammatory cells, and stromovascular and immunological cells. Its proximity to the cardiac tissue allows for direct interaction and crosstalk between epicardial fat and the myocardium. EAT can lead to the development and progression of coronary artery disease, atrial fibrillation, and heart failure [123,124]. Generally, EAT is considered white adipose tissue, but it presents similarities with brown fat, since uncoupling protein 1 (UCP-1), a marker of brown adipose tissue, is highly expressed in EAT. Moreover, EAT is an active endocrine organ that secrets numerous adipokines, including adiponectin, leptin, omentin-1, and a series of inflammatory factors. Through the paracrine pathway, the released molecules can be directly diffused into the underlying myocardium and coronary arteries. EAT from patients with CVD expresses high levels of pro-inflammatory biomarkers such as TNF-α, IL-6, and IL-1ß [125,126]. Dysregulated EAT releases hypoxia-inducible factor 1-alpha (HIF-1α), potentiating myocardial nicotinamide adenine dinucleotide phosphate (NADPH) oxidase activity and ROS production. ROS promotes cardiac fibrosis and hypertrophy through the Akt/mTOR/Nuclear factor kappa B (NF-kB) pathways, leading to the development of heart failure. The EAT secretome determines the site of heart inflammation by releasing a variety of pro-inflammatory adipokines, such as leptin, IL-6, TNF-α, visfatin, omentin, resistin, and anti-inflammatory factors like adrenomedullin and adiponectin. Pro-inflammatory cytokines drive EAT-derived mesenchymal stem cells into the myocardium, transforming them into fibroblasts. Thus, the adipokines secreted by EAT could regulate the process of myocardial fibrosis [127,128,129]. In summary, when secretion is dysregulated, inflammation and OxS occur, facilitating the development of CVD. Therefore, in cases associated with abnormal glucose and lipid metabolism, atherosclerosis, hypertension, myocardial hypertrophy, heart failure, and even myocardial infarction may occur [23,130,131,132]. The pathogenesis related to atherosclerosis is closely related to metabolic disorders. For several years, a tight connection with non-alcoholic fatty liver disease related to metabolism (MAFLD) has been observed. This condition, linked to insulin resistance, overweight/obesity, T2DM, and atherogenic dyslipidemia, i.e., cardiometabolic disorders, intertwines changes involving the brain–gut–liver axis, consequently affecting other organs indirectly, such as the heart [133,134]. Atherosclerosis, a chronic inflammatory condition that affects medium and large arteries, is characterized by the development of intimal plaque, thrombosis, and stenosis of the vessel lumen, and involves the rupture of the endothelium, the activation of the inflammatory cascade, the migration of monocytes to the media layer, the proliferation of smooth muscle cells, and the formation of atheromatous plaques, initially causing a decrease in blood flow and then hypoxia. Elevated glycemia and the presence of dyslipidemia increase the risks of atherosclerosis and plaque necrosis through several signaling pathways, such as the prolonged increase in ROS and inflammatory factors in cardiovascular cells, currently the leading cause of heart attacks, stroke, and vascular diseases [135,136,137].

Furthermore, atherogenesis occurs in areas where laminar blood flow is disturbed and shear stress is altered. Consequently, lipids and immune cells penetrate the subendothelial layer in the vessel wall, and LDL-c can be oxidized by ROS and oxLDL, stimulating inflammation. In this process, adhesion molecules are expressed, causing monocytes to adhere and transform into macrophages that phagocytose oxLDL. Macrophages transform into foam cells, secreting inflammatory cytokines and chemokines and recruiting new cells (monocytes and T lymphocytes). With this cellular, lipid, and fat aggregation, the formation of increasingly complex plaques occurs [138,139,140,141].

IL-1 family cytokines (mainly IL-1β and IL-18) are crucial to the vascular and systemic inflammation process related to atherosclerosis development. On the other hand, the synthesis of these mediators is directly linked to NLRP3 inflammasome. The p38 mitogen-activated protein kinase (p38δ MAPK) is an NLRP3 inflammasome regulator, and its activation is associated with increased coronary atherogenesis. IL-1 can interfere with many cells involved in the pathogenesis of atherosclerosis. It can enhance endothelial cell barrier permeability, attracting monocytes into the vessel wall and resulting in smooth muscle cell lesions and atherosclerosis [142,143,144].

TLRs are well-known receptors involved in the immune system. TLR-4 is related to fatty acid-induced lipid accumulation and can trigger inflammatory responses in the heart. TLR-7 can be expressed in platelets, endothelial cells, and vascular smooth muscle cells, stimulating the production of inflammatory (such as IL-1, IL-6, IL-12, and TNF-α) and anti-inflammatory cytokines (such as IL-10), which are related to atherosclerosis [8,145,146,147,148].

Inflammation and OxS are also involved in the pathogenesis of Rheumatic Heart Disease [149]. In rheumatic diseases, the inflammatory condition facilitated by the release of IL-6, IL-8, TNF-α, and Interferon-γ (IFN-γ) by macrophages and the synthesis of autoantibodies that induce the formation of vascular cell adhesion molecule (VCAM1) potentiates endothelial dysfunction. Additionally, the combination of reduced nitric oxide (NO) bioavailability and increased ROS production by nicotinamide adenine dinucleotide phosphate (NADPH) oxidase promotes the progression of this condition [150,151,152,153,154].

The pathogenesis of atrial fibrillation (AF) involves an interaction between the inflammatory response, OxS, and the development of atrial fibrosis. Galectin-3 (Gal-3) is a lectin family protein involved in cell differentiation, fibrinogenesis, and inflammation. Gal-3 activates (myo)fibroblasts and endocardial cells, inducing fibrogenesis by increasing the extracellular matrix (ECM). Myeloperoxidase (MPO), released by neutrophils, plays a significant role in the interaction between inflammation and OxS, leading to cardiac fibrosis [155,156,157,158].

**Figure 4:**
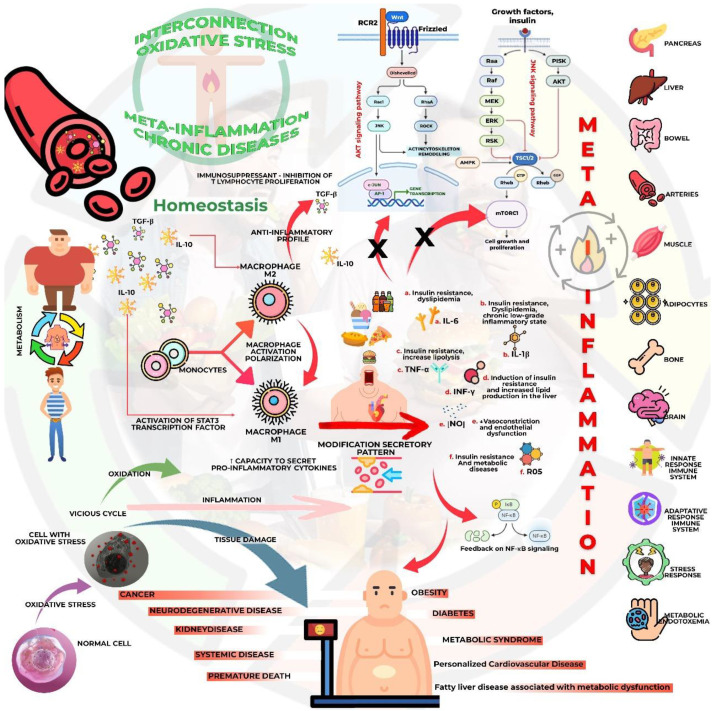
Interconnections between oxidative stress and inflammation. The persistent and systemic low-grade inflammation known as metabolic inflammation or metainflammation plays a fundamental role in the pathogenesis and aggravation of metabolic diseases, such as diabetes and cardiovascular diseases. The principal mechanism is through the disfunction of cytokines with an excess of her productions. The changes in the secretory pattern can worsen glycolipid disorders, and finally, 1314 maintains the metainflammatory metabolism. IL-1β: Interleukine-1β; IL-6: Interleukine1315 6; IL-10: Interleukine-10; INF-γ: Interferon-γ; NF-kβ: Nuclear factor kappa β; NO: nitric oxide; ROS: reactive oxygen species; TGF-β: Transforming Growing Factor-β; 1317 TNF-α: tumor necrosis factor-α.

Inflammation and OxS are involved in the problematic outcomes of acute myocardial infarction (AMI). The MAPK signaling pathway and the NF-kB signaling pathway play an essential role in the secretion and activity of TNF-α, IL-2, IL-1, and other pro-inflammatory cytokines in human endothelial cells within the context of inflammation. On the oxidative side, levels of AMI are significantly elevated, which can cause dysfunction and cellular damage in the myocardium [159,160,161,162]. Figure 4 summarizes the relationship between inflammation and OxS.

## 6. The Role of mTOR

The mechanistic target of rapamycin (mTOR) plays a central role in regulating cellular growth and metabolism in complex physiological processes. This molecule utilizes the availability of energy and nutrients to regulate its cellular activities, such as cell proliferation, cell growth, protein synthesis, and autophagy [163]. Depending on the metabolic pathway that activates this molecule, mTORC1 responds by acting on different substrates. In this sense, it can promote anabolism, the formation of proteins, nucleic acids, and lipogenesis. It can also regulate cellular metabolism, controlling energy expenditure. Additionally, it can inhibit catabolic processes, including autophagy. Specifically in lipid metabolism, mTORC1 controls the synthesis of lipids and nucleic acids through the transcription factor sterol regulatory element-binding protein 1/2 (SREBP1/2), a substrate that regulates the expression of multiple lipid genes. Regarding protein synthesis, mTORC1 regulates the synthesis of new pyrimidines and purines in various cellular models to enrich the nucleotide pool for nucleic acid synthesis, essential for maintaining DNA replication and RNA synthesis [164].

However, the dysregulation of mTOR signaling has negative implications, as it may be associated with many diseases such as cancer, diabetes, cardiovascular disease, and neurological diseases. Other cellular stresses, such as amino acid deficiency, hyperosmolarity, and pH stress, negatively regulate mTORC1 [165]. mTOR forms part of an extensive network of signaling pathways crucially involved in various human diseases. Positioned downstream of phosphatidylinositol 3-kinase (PI3K), this network includes essential components such as phosphoinositide-dependent kinase 1 (PDK1), protein kinase B (Akt), serum/glucocorticoid-regulated kinase 1 (SGK1), and AMP-activated protein kinase (AMPK), all contributing to the diverse functions of mTOR [166]. In diabetes, mTOR has been shown to play a key role in affecting insulin resistance and sensitivity, glucose uptake, lipid metabolism, and ketone production [167]. In cardiology, prolonged hyperactivation of mTOR signaling in diabetes worsens post-ischemic myocardial injuries by accelerating cardiomyocyte death, leading to cardiac remodeling and inflammatory responses [168]. Approximately 30% of cancers utilize the mTOR signaling pathway, contributing significantly to their development and advancement by influencing cell cycle regulation, growth, survival, and metabolism. Moreover, it plays a pivotal role in regulating nutrient utilization and energy production, underscoring its critical metabolic function in facilitating cancer progression and invasiveness, including processes like angiogenesis and metastasis. The dysregulation of key cancer-associated genes results in mTOR hyperactivation, enhancing the synthesis of pro-oncogenic proteins that directly impact cellular functions such as proliferation, migration, and angiogenesis. Multiple studies suggest that mTOR signaling plays a crucial role in both normal wound healing and the development of pathological fibrosis. Dysregulated mTOR signaling contributes to fibrotic diseases by promoting fibroblast proliferation, TGF-β1-induced myofibroblast differentiation, excessive extracellular matrix (ECM) accumulation, and increased collagen production. These processes can exacerbate a range of vascular, cardiac, and other diseases [169].

## 7. GLMD and Metainflammation: A Systemic Imbalance

The persistent and systemic low-grade inflammation known as metabolic inflammation or metainflammation is derived from dysregulations in lipid and glucose metabolism, leading to hypertrophy/hyperplasia of the adipose tissue [170,171]. Metainflammation can occur due to the dysfunction of the immune system caused by GLMD (Figure 4), impairing the proper functioning of the immune system and promoting chronic non-infectious inflammation that plays a fundamental role in the pathogenesis and aggravation of metabolic diseases, such as T2DM and CVD. It is strongly related to OxS mechanisms, occurring in a bidirectional manner, meaning that inflammation induces OxS and OxS induces inflammation [172,173,174].

Disorders in adipose tissue homeostasis result in adipocyte hypertrophy, with excess nutrients and the persistence of chronic low-grade systemic inflammation (CLSI) leading to the functional impairment of immune cells and/or unbalanced cytokine production. In this context, protein 3 (PTX3) is an important mediator in linking obesity, CLSI, and innate immunity, as it is produced by inflammatory pathways induced by TNF-α and IL-1β, strongly related to the metainflammatory state, as this protein has a high potential for lipid accumulation. Furthermore, hyperlipidemia is partly induced by macrophages’ inefficient action in maintaining tissue homeostasis in adipose tissue. Therefore, an increased risk indicator of CVD is perceived since these pro-inflammatory cytokines can occur adjacent to cardiac tissue [175,176,177,178].

As already mentioned, atherogenesis results from alterations in intracellular metabolic pathways of the arterial wall, including increased glycosylation, clearly indicating the vascular inflammatory level. In this pro-inflammatory environment, immune cells adapt to various environmental signals and biosynthetic requirements, but when their function is altered, there are vascular damage [179,180,181].

It is known that metainflammation is closely related to ROS. The main mediators activated in this relationship are hypoxia-inducible factor 1-alpha (HIF-1α) and NF-kB, which induce the transcription of several inflammatory mediators, including CC motif chemokine ligand 2/monocyte chemoattractant protein-1 (CCL2/MCP-1), CXC motif chemokine ligand 1/growth-regulated oncogene-alpha (CXCL1/GRO-α), CXC motif chemokine ligand 8/interleukin 8 (CXCL8/IL-8), and cyclooxygenase-2 (COX-2) together with PGE2 (prostaglandin E2) [182]. Additionally, in poorly vascularized adipose tissue, the activation of NF-ΚB and hypoxia-inducible factor 1-alpha (HIF-1α) is responsible for the positive regulation of TNFα, IL-1, IL-6, matrix metalloproteinases (MMP) 9 and MMP2, monocyte chemoattractant protein-1 (MCP-1), plasminogen activator inhibitor-1, macrophage migration inhibitory factor, and inducible NO synthase [183,184]. These factors reorganize the immunological compartment of the inflammatory microenvironment through multiple mechanisms, thus promoting the enrollment of neutrophils, macrophages, and other immune cells potentially implicated in the progression of possible CVDs [185,186,187,188,189].

After the ingestion of energy-rich foods with a low nutrient content, remnants of chylomicrons and very low-density lipoproteins (VLDLs), triglyceride-rich lipoproteins, bind to endothelial cells and leukocytes present in the blood, causing an increase in the release of adhesion molecules, cytokines, and OxS species, such as thiobarbituric acid reactive substances (TBARSs), O^2−^ leukocytes, and 8-iso-prostaglandin F2alpha (8PGF2α). VLDLs and free fatty acids (FFAs) enhance the expression of VCAM1 in endothelial cells of the human aorta, favoring the adhesion of defense cells. This inflammatory environment promotes IR through NF-κB and c-Jun N-terminal kinase (JNK) pathways, leading to a decline in insulin secretion by pancreatic β-cells. This complex system can contribute to the development of atherogenesis [190,191].

Other circulating inflammatory and metabolic biomarkers, including CRP, TNFα, fasting blood glucose, fasting insulin, and lipid profiles, are important predictors of cardiometabolic risk related to metainflammation [190,192,193,194].

## 8. GLMD and Cardiovascular Diseases: The Final Connection

**Figure 5:**
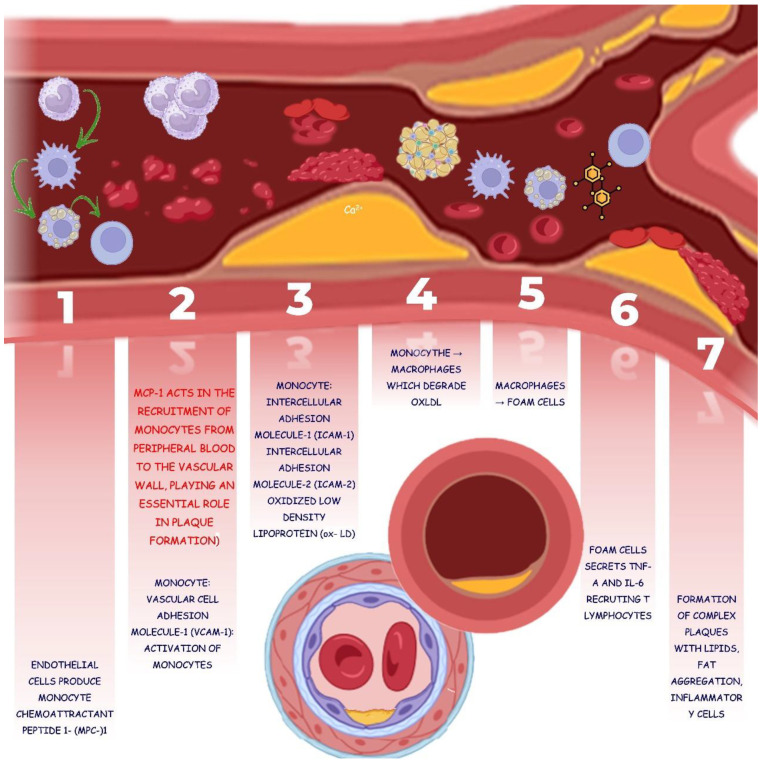
The physiopathology of atheroma’s plaque is closely associated with glucolipid metabolic disorders. Increased circulating lipids and oxidation promoted by free radicals can initiate plaque formation. Endothelial dysfunction occurs and oxLDL-c are recognized and phagocytosed by macrophages, leading to the formation of foam cells. The next steps are related to the initiation and progression of the atherosclerotic plaque that can occlude the arteries. ICAM-1: intercellular adhesion molecule-1; ICAM-2: intercellular adhesion molecule-2; IL-6: Interleukin-6; ox-LDL: oxidized low-density lipoprotein; MPC-1: monocyte chemoattractant peptide-1; TNF-α: tumor necrosis 1327 factor-α; VCAM-1: vascular cell adhesion molecule-1.

**Figure 6:**
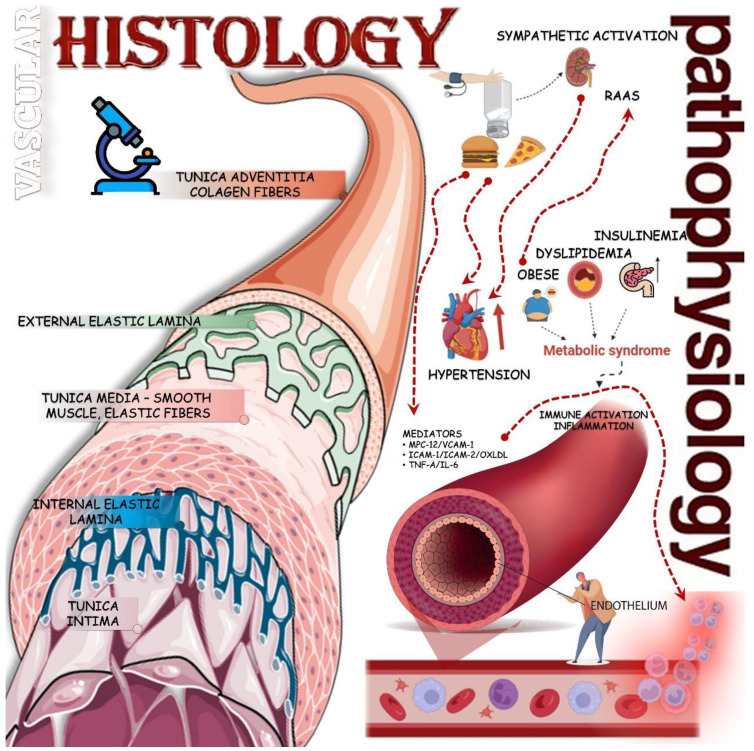
Higher chronic consumption of sodium promotes malefic changes in the nervous system and many alterations in the vascular endothelium. Firstly, the activation of the renin–angiotensin–aldosterone system occurs to equal the osmotic concentration in the blood increasing the blood pressure. Furthermore, the unhealthy food habits promote issues in vascular tunica intima, leading to the secretion and accumulation of inflammatory cells and cytokines in the vessels, resulting in atherosclerosis. In addition, the higher lipid concentration creates difficulty for the signaling of insulin receptors, promoting insulin resistance. Finally, all of these changes are connected with metabolic syndrome and other cardiovascular issues. ICAM-1: intercellular adhesion molecule-1; ICAM-2: intercellular adhesion molecule-2; IL-6: Interleukin-6; MPC-12: monocyte chemoattractant peptide-12; ox-LDL: oxidized low-density lipoprotein; TNF-α: tumor necrosis factor-α; VCAM-1: vascular cell adhesion molecule-1.

GLMD is a complex condition whose main pathological mechanisms are neuroendocrine dysregulation, insulin resistance, OxS, metainflammation, and dysbiosis, all of which are associated with lifestyle and can lead to nutritional deficiencies, cellular organic imbalance, the accumulation of harmful species in the bloodstream, and imbalanced cellular signaling, ultimately culminating in life-threatening diseases mainly affecting vital organs such as the heart [195,196]. Thus, the main clinical manifestations are hyperglycemia, dyslipidemia, hepatic steatosis, and atherosclerosis, indicating a scenario of complex interconnection and numerous co-occurring aggressions. In summary, GLMD leads to the emergence of inflammatory factors and dysregulated immune cells, putting interconnected tissues and organs at risk, mainly by promoting alterations in blood vessels, pancreatic islets, the liver, and adipocytes, forming a network of crosstalk through inflammatory mediators and signaling pathways to influence disease development (Figure 5 and Figure 6) [8,197,198,199,200].

Elevated LDL-c levels are associated with a higher risk of sudden cardiac arrest (SCA) [201,202]. LDL-c dissociates from the LDL receptor within the hepatocyte and undergoes endosomal degradation. In contrast, the receptor can return to the cell surface and continue facilitating the removal of LDL-c from circulation. The proprotein convertase subtilisin/kexin type 9 (PCSK9) binds to the LDL particle/LDL receptor complex, preventing intracellular dissociation within the hepatocyte and thereby limiting the hepatic uptake of LDL particles [203,204]. Therefore, the full functionality of PCSK9 may be related to elevated blood LDL-c levels and, subsequently, the development of hypercholesterolemia, which can result in CVD [199,205,206,207].

Endothelial dysfunction, in addition to the action of AGEs, is induced by the positive regulation of biomolecules such as vascular endothelial growth factor and plasminogen activator inhibitor-1 (PAI-1), leading to neoangiogenesis and increasing the pro-thrombotic state essential for the development of CVD [17,18]. AGE-RAGE binding triggers various intracellular reactions, resulting in the production and release of pro-inflammatory cytokines such as IL-6, TNF-α, TGF-β, VCAM-1, ICAM-1, and ET-1, which also have a close relationship with CVD 19–22 (Figure 5 and Figure 6).

Endothelial cells are crucial in preserving vascular integrity and ensuring appropriate blood flow. The healthy tissue efficiently organizes blood vessels, including arterioles, capillaries, and venules [208]. However, the vascular lining is particularly susceptible to harmful stimuli that trigger tumor-suppressive pathways, leading to cellular senescence recognized as a significant factor in various cardiovascular and metabolic diseases due to a blend of multiple mechanisms, including compromised coronary perfusion, cardiac dysfunction in systole/diastole, microvascular injury, and abnormal hemodynamics in the arterial tree [209,210]. Under conditions of OxS, changes in glycolipids within biological membranes can be implicated in various pathological states, including atherosclerosis, neurodegeneration, carcinogenesis, glucose intolerance, and dyslipidemia [211,212].

Excessive lipid accumulation contributes to glucose intolerance and dyslipidemia; uncontrolled glycolipid metabolism in T2DM can damage blood vessels through the inability of endothelial cells to maintain vascular homeostasis, the formation of AGEs, OxS, inflammation, and epigenetic modification. This scenario occurs in several organs, notably the kidneys, eyes, heart, and nerves, with vascular complications such as diabetic neuropathy, diabetic retinopathy, diabetic nephropathy, myocardial infarction, and stroke [213,214,215].

The hyperglycemic state involved in GLMD pathology promotes aggression in the glomeruli by altering the polarity of filtration barriers, affecting podocytes and the glomerular filtration rate, leading to the emergence of proteinuria and disrupting the hydroelectrolytic balance between blood and urine. Thus, it dysregulates the juxtaglomerular apparatus, consequently causing dysfunction of the renin–angiotensin–aldosterone system (RAAS) with hemodynamic and cardiologic impairments [216,217,218]. Similarly, the alteration in metabolic glycolipid order assists in the formation of pro-inflammatory species such as TNF-α, IL-6, hs-CRP, IFN-γ, and monocyte chemoattractant protein-1. All of these molecules actively participate as inflammatory messengers triggering aggressive events in various locations of the body, including the cardiac microenvironment during pericarditis, myocardial infarction, cardiac hypertrophy, cardiac tamponade, coronary artery disease, and others [219,220].

Growth differentiation factor 15 (GDF-15) is a transforming growth factor β TGF-β) superfamily member and has been significantly correlated with GLMD and CVDd. The adipose tissue secretes GDF-15, which functions as an adipokine and may have a paracrine role in regulating adipose tissue function and body mass [221]. It is a cytokine produced in multiple pathological processes in response to tissue injury and inflammatory states by cardiomyocytes, adipocytes, macrophages, endothelial cells, and vascular smooth muscle cells, including obesity, diabetes, metabolic syndrome, heart failure, atherosclerosis, inflammatory diseases, pulmonary hypertension, acute coronary syndrome, myocardial infarction, ischemia reperfusion injury, diabetic cardiomyopathy, and atrial fibrillation [222]. Under these conditions, GDF-15 is secreted, where it plays a tissue protective role through the positive and negative regulation of several signaling pathways; therefore, its higher serum concentrations may be a clinically relevant biomarker in the context of metabolic syndrome and the development of pathological cardiac conditions. The aging process and lifestyle sustain and collaborate with the progress of chronic inflammation related to cellular and tissue dysfunction. Although GDF-15’s effects are tissue-specific and are also dependent on microenvironmental modifications such as inflammation and OxS, some studies suggest that it has a significant role in CVD since it has an important regulatory role in the development and maintenance of atherosclerosis, one of the major causes of CVD [223,224,225,226].

## 9. Important Acknowledgments to Improve the Treatment of Metabolic Diseases and Cardiovascular Diseases

Despite the vast amount of available knowledge, many unknown paths exist to improve the treatment of existing diseases. A detailed understanding of pathophysiological processes plays a crucial role in advancing new therapies, aiming not only to prevent the development of these diseases but also to halt their progression. In the specific case of diabetes, the complexity of the pathophysiological processes presents multiple facets. So far, traditional approaches such as lifestyle changes, oral medications, and injectable insulin have not yielded satisfactory results, exacerbated by an incomplete understanding of diabetes pathogenesis. Targeted therapies involving DNA and RNA are emerging as promising avenues to personalize treatment and accurately diagnose and potentially prevent diseases. For example, RNA-based therapies use ribonucleic acid molecules to modulate protein expression, offering new perspectives to prolong patient survival, improve treatment effectiveness, and pave the way for future research in diabetes treatment. These innovations represent a promising direction for developing more effective treatments, underscoring the importance of a deep molecular understanding of diseases to revolutionize therapeutic approaches [227].

The excess accumulation of triglycerides in adipose tissue can lead to adipose dysfunction, inflammation, and lipotoxicity in non-adipose tissues. These conditions are closely linked to obesity and glucose regulation in the body, influenced by mediators such as ceramide and sphingosine-1-phosphate (S1P), crucial sphingolipids. Ceramide, when accumulated in cells, can impair the survival of pancreatic β cells and promote insulin resistance in the liver and skeletal muscle. Conversely, S1P, depending on the activation pathway (especially through subunits S1P1, S1P2, and S1P3), can influence different patterns of cellular expression and intracellular targets. Research indicates that activating S1P1 or S1P3 subunits may improve obesity and associated metabolic disorders, such as cardiovascular events. In contrast, the activation of S1P2 has been associated with opposing adverse effects. Therefore, understanding these interactions among sphingolipids, obesity, and glucose regulation is crucial for developing new treatments aimed at mitigating the negative effects of metabolic dysfunction and opening new therapeutic perspectives in managing obesity and its metabolic complications [228].

In addition to understanding chemical mediators, receptors, and their potential interactions, it is crucial to comprehend the fundamentals of microvascular homeostasis to develop new therapeutic strategies aimed at reducing cardiovascular morbidity and mortality. Ceramide, for instance, is recognized for its adverse effects on the microvasculature, increasing the risk of severe cardiac events when found at elevated levels in plasma. Therefore, clarifying its metabolic functions and understanding its blood concentrations are essential steps toward developing innovative treatments in this field [229,230].

Finally, by detailing numerous types of molecules and their potential functions, we facilitate an understanding of their roles in the human body, thereby creating new therapeutic possibilities.

## 10. Conclusions and Future Perspectives

Adipose tissue is essential for regulating lipid and glucose homeostasis. GLMD causes excess fatty adipose tissue, which is essential for regulating lipid and glucose homeostasis. GLMD causes excess fat to spill into the blood through non-esterified fatty acids, affecting several organs such as the skeletal muscle, heart, liver, kidneys, and pancreas. This can lead to insulin resistance, abnormal lipid deposits, lipid metabolism dysregulation, and the start of inflammatory and oxidative processes in a vicious cycle. The consequences of these events not only affect the cardiac system, but the repercussions are systemic and will cause organ damage and increased morbidity and mortality.

Research initiatives could dissect the intricate interactions between glycolipids and inflammatory pathways to uncover their roles in immune modulation and disease pathogenesis. By employing cutting-edge techniques such as lipidomics and high-resolution imaging, researchers could elucidate the molecular mechanisms underlying glycolipid-mediated immune modulation, pinpointing key signaling events and cellular targets. Furthermore, exploring the impact of glycolipid metabolism dysregulation in various inflammatory conditions, such as autoimmune diseases or chronic inflammatory disorders, may provide valuable insights into disease pathways and identify potential therapeutical targets. Additionally, investigating the therapeutic potential of targeting glycolipid pathways or manipulating glycolipid metabolism could open new avenues for the development of precision medicines aimed at modulating immune responses and mitigating inflammatory diseases.

Other research initiatives could also focus on leveraging advanced imaging technologies to unravel the complexities of atherosclerotic plaques and their microenvironments within the vasculature.

Furthermore, research efforts could center on unraveling the multifaceted role of S1P in cardiovascular health and disease, delving into its intricate signaling pathways and interactions within the vascular microenvironment. By elucidating the specific receptors and downstream effectors involved in S1P-mediated responses, researchers could uncover novel therapeutic targets for mitigating cardiovascular pathologies and inflammation-related disorders. Furthermore, exploring the potential of S1P modulation in regulating endothelial function, immune cell behavior, and smooth muscle cell phenotypes may offer insights into its therapeutic potential for maintaining vascular homeostasis and preventing atherosclerosis progression.

Research endeavors could leverage single-cell sequencing technologies to comprehensively characterize the diverse cellular populations present within atherosclerotic plaques. By dissecting individual cells’ molecular signatures and functional states, it will be possible to uncover key players driving plaque biology and disease progression. Furthermore, investigating the influence of environmental factors, such as diet, microbiota, and systemic inflammation, on plaque composition and cellular behavior could unveil novel therapeutic targets and inform personalized interventions for cardiovascular disease management.

## Data Availability

No new data were created or analyzed in this study. Data sharing is not applicable to this article.

## Abbreviation

Adipose tissue (AT); acute myocardial infarction (AMI); advanced glycation end products (AGEs); Angiopoietin-like protein (ANGPTL); Angiotensin II (Ang II); Angiotensin-converting enzyme 2 (ACE2); atrial fibrillation (AF); ATP-binding cassette transporter 1 (ABCA1); cardiorenal vascular disease (CRVD); CC motif chemokine ligand 2/monocyte chemoattractant protein-1 (CCL2/MCP-1); chronic low-grade systemic inflammation (CLSI); c-Jun N-terminal kinase (JNK); copper deficiency (CuD); coronary artery disease (CAD); C-reactive protein (CRP); Cyclooxygenase-2 (COX-2); Cytosolic phospholipase A2 (cPLA2); CXC motif chemokine ligand 1/growth-regulated oncogene-alpha (CXCL1/GRO-α); electron transport chain (ETC); endothelin-1 (ET-1); epicardial adipose tissue (EAT); extracellular matrix (ECM); Fibroblast growth factor 21 (FGF-21); free fatty acids (FFAs); Galectin-3 (Gal-3); Glycated hemoglobin (HbA1c); glycolipid metabolic disorder (GLMD); Glucagon-like peptide-1 (GLP-1); Glucose transporter 4 (GLUT4); growth differentiation factor 15 (GDF-15); high-density lipoprotein (HDL-c); hydrogen peroxide (H_2_O_2_); hypoxia-inducible factor 1-alpha (HIF-1α); insulin resistance (IR); intercellular adhesion molecule-1 (ICAM-1); Interferon-γ (IFN-γ); Interleukin (IL)-6; Leucocyte cell-derived chemotaxin 2 (LECT2); lipopolysaccharide (LPS); low-density lipoprotein (LDL-c); matrix metalloproteinases (MMPs); metabolic-associated fatty liver disease (MAFLD); monocyte chemoattractant protein-1 (MCP-1); Nicotinamide adenine dinucleotide phosphate (NADPH); Nicotinamide adenine dinucleotide phosphate oxidases (NOXs); nitric oxide (NO); Nuclear factor kappa B (NF-kB); oxidative stress (OxS); oxidized LDL-c (ox-LDL); peroxisome proliferator-activated receptors (PPARs); plasminogen activator inhibitor-1 (PAI-1); Proprotein convertase subtilisin/kexin type 9 (PCSK9); Prostaglandin E2 (PGE2); protein 3 (PTX3); protein kinase C (PKC); p38 mitogen-activated protein kinase (p38δ MAPK); reactive oxygen species (ROS); renin–angiotensin–aldosterone system (RAAS); sarcoplasmic/endoplasmic reticulum Ca^2+^ ATPase 2a (SERCA2a); sphingosine-1-phosphate (S1P); sudden cardiac arrest (SCA); superoxide anion (O^2−^); Thiobarbituric acid reactive substance (TBARS); Toll-like receptor-4 (TLR-4); Transforming Growing Factor (TGF)-β; triglycerides (TGs); tumor necrosis factor-α (TNF-α); Type 2 Diabetes Mellitus (T2DM); uncoupling protein 1 (UCP-1); vascular adhesion molecules like (VCAM-1); vascular non-inflammatory molecule-1 (VNN1); very low-density lipoprotein (VLDL); 8-iso-prostaglandin F2alpha (8PGF2α).
